# Indigenous oral traditions and psychological well-being under modernization: from external valuation to relational selfhood

**DOI:** 10.3389/fpsyg.2026.1912239

**Published:** 2026-08-06

**Authors:** Ting-Ting Liu, Jun-Yi Chen

**Affiliations:** 1 Chengdu University of Technology, Chengdu, China; 2 Fuzhou University of International Studies and Trade, Fuzhou, China; 3 Research Center for the Inheritance and Innovation of Handicraft Culture of Humanities and Social Science Base of Fujian Province Universities, Fuzhou, China

**Keywords:** cultural continuity, decolonial psychology, external valuation, indigenous oral traditions, indigenous psychological well-being, meaning-making, relational selfhood, traditional knowledge

## Abstract

Across many Indigenous communities, modernization, urbanization, market integration, formal schooling, language shift, and cultural commodification have reshaped the conditions under which psychological well-being is understood and sustained. These pressures influence how Indigenous identities, languages, knowledges, and cultural practices are valued within dominant social, educational, economic, and clinical frameworks. This article conceptualizes Indigenous oral traditions as living psychosocial knowledge embedded in community-held epistemic and relational infrastructures and examines their significance for psychological well-being under modernization. Drawing on Indigenous and decolonial perspectives, it introduces external valuation as a middle-range analytical construct describing how Indigenous lifeworlds are judged through criteria defined outside the communities concerned, including productivity, marketability, standardized achievement, formal recognition, policy usefulness, and public visibility. Such valuation may intensify alienation, shame, cultural disconnection, identity insecurity, weakened belonging, and loss of self-continuity. Oral traditions, including storytelling, creation accounts and sacred histories, songs, chants, ritual narratives, oral histories, and place-based accounts, are examined as practices that carry memory, ethics, identity, spiritual orientation, and intergenerational knowledge. The article proposes four interrelated psychosocial pathways through which oral traditions may become relevant to culturally grounded well-being: meaning-making, cultural continuity, relational selfhood, and social cohesion. These pathways are integrated into a conceptual model linking modernization pressures, external valuation, psychosocial risks, oral traditions, and psychological well-being. By distinguishing broader empirical support, context-specific evidence, and theoretically informed inference, the article avoids presenting these relations as established causal effects. It concludes with implications for culturally inclusive mental health initiatives grounded in community authority, relational accountability, local protocols, and respect for Indigenous knowledge systems.

## 1 Introduction

Psychological well-being is often discussed in mainstream psychology through the language of individual functioning, adjustment, coping, resilience, and symptom reduction. These concepts remain important, but they do not fully account for the ways well-being is understood and sustained in many Indigenous communities. In Indigenous psychological and social–emotional frameworks, well-being is rarely confined to an internal state of the individual. It is more often situated in relations with land, family, community, language, spirituality, ancestry, cultural practice, and collective memory. The issue, therefore, is not simply that Indigenous communities have culturally specific expressions of mental health. Rather, Indigenous perspectives ask whether the conceptual foundation of psychological well-being itself needs to be reconsidered.

This question has become increasingly central in Indigenous psychology, community psychology, and social and emotional well-being research. Across different Indigenous contexts, scholars have challenged the assumption that psychological well-being can be adequately defined through universalized, individual-centered models. Indigenous psychological paradigms emphasize relationality, interdependence, cultural continuity, and ecological embeddedness, while decolonial approaches remind us that psychological knowledge is always shaped by histories of power, place, and epistemic authority (Blume, 2022; Ciofalo et al., 2022; Milroy et al., 2023). In this sense, culture is not an additional variable to be added to an otherwise complete psychological model. It is part of the ground through which selfhood, distress, healing, and belonging become intelligible.

The limitations of mainstream psychological models become especially visible when they are applied to Indigenous communities without sufficient attention to cultural and historical context. Models that prioritize individual autonomy, self-management, internal emotional regulation, or clinical symptom categories may capture some dimensions of psychological life, but they can overlook the collective and relational conditions through which suffering and healing are experienced. Work on Aboriginal and Torres Strait Islander health, for example, has shown that even well-established therapeutic frameworks require careful reconsideration when used in Indigenous contexts (Kilcullen et al., 2016). Similarly, reviews of Indigenous community-based mental wellness initiatives suggest that effective mental wellness practices are often inseparable from community grounding, cultural relevance, and Indigenous authority (Dudgeon et al., 2021; Venugopal et al., 2021). The problem is not that mainstream models are always irrelevant, but that they become partial when they treat Indigenous well-being as a culturally modified version of an already settled psychological framework.

A relational understanding of well-being also requires psychology to take place seriously. In many Indigenous contexts, land is not merely a physical environment or a background setting for human life. It may carry memory, obligation, spirituality, identity, and belonging. Research on Māori well-being has emphasized the cultural and environmental specificity of wellness, while recent work on decolonizing the psyche has similarly foregrounded the interconnection of land, spirituality, and well-being (Panelli and Tipa, 2007; Renfrew and Hamley, 2025). These perspectives complicate psychological models that detach well-being from place. If well-being is partly formed through relations with ancestral land, ecological worlds, and place-based memory, then psychological distress cannot be fully understood as an individual condition alone.

Language and cultural practice further illustrate this point. Studies on Indigenous language and well-being show that language is not only a communicative instrument. It may also carry relationships with ancestors, community, spirituality, and place. The reclamation of Barngarla, for instance, has been associated with community understandings of well-being, while research on Indigenous language use has linked language, spiritual connectedness, and positive mental health (Gonzalez et al., 2021; Sivak et al., 2019). These studies are significant because they show that cultural continuity is not simply a matter of preserving heritage. It can shape how people experience identity, connection, and psychological security.

Within this broader relational framework, Indigenous oral traditions deserve closer conceptual attention. Storytelling, creation accounts and sacred histories, songs, chants, ritual narratives, ancestral stories, and place-based narratives are often described as cultural heritage or intergenerational knowledge transmission. This description is accurate but insufficient. Oral traditions do preserve knowledge, yet they also organize memory, transmit moral and spiritual orientations, sustain collective identity, and offer ways of interpreting suffering, loss, belonging, and continuity. Scholarship on Indigenous oral traditions has emphasized their role in intergenerational transmission, while phenomenological work with Indigenous populations has drawn attention to oral tradition as a way of understanding time, memory, and experience (Mittal et al., 2025; Mukuni, 2025; Struthers and Peden-McAlpine, 2005). Oral traditions, then, are not simply remnants of the past. They are living practices through which communities continue to make sense of the present.

This point is important for psychological well-being because oral traditions may help sustain the conditions through which well-being becomes possible. They can provide narrative forms for grief, displacement, historical injury, and cultural loss. They can also connect individuals to ancestors, land, language, kinship, and shared memory. In related psychological and peacebuilding literature, storytelling has been discussed in connection with counseling, social repair, and the organization of collective meaning (Coburn, 2012; Jenkins, 2013). Such work suggests that narrative practices can participate in meaning-making, emotional articulation, relational repair, and community belonging, with their significance shaped by cultural context and collective identity. In Indigenous contexts, these processes are often inseparable from cultural continuity and collective identity.

This article conceptualizes Indigenous oral traditions as living psychosocial knowledge whose relevance to psychological well-being can be examined through four interrelated pathways: meaning-making, cultural continuity, relational selfhood, and social cohesion. Oral traditions are approached as culturally situated practices through which communities organize memory, interpretation, identity, ethical orientation, intergenerational relations, and belonging. Their contemporary significance arises from the community-held relations through which they are authorized, performed, interpreted, and renewed.

The central question of this article is therefore: How do Indigenous oral traditions retain contemporary psychosocial significance amid modernization, and through what conceptual pathways may they become relevant to culturally grounded psychological well-being? To address this question, the article develops three linked arguments. First, Indigenous psychological well-being is relational and culturally grounded, encompassing connections with land, language, ancestry, spirituality, family, community, and collective memory. Second, oral traditions function as living psychosocial knowledge that organizes meaning, memory, identity, ethical orientation, and intergenerational relations. Third, their contemporary significance lies in their capacity to sustain meaning, continuity, belonging, and relational self-understanding under conditions of external valuation.

This article undertakes a cross-contextual, purposive conceptual analysis of scholarship concerning specific Indigenous peoples and communities. It identifies recurring psychosocial relations across studies while interpreting each source in relation to the people, place, language, historical conditions, and oral practice it addresses. The synthesis distinguishes community-specific studies, broader Indigenous well-being research, and adjacent conceptual scholarship. Section 2 explains how the literature was identified, categorized, evaluated, and integrated.

## 2 Methodological approach to the conceptual analysis

This study used a purposive conceptual analysis to examine the relations among modernization pressures, external valuation, Indigenous oral traditions, psychosocial processes, and culturally grounded psychological well-being. The analysis brought together work from Indigenous psychology, mental health and well-being research, oral tradition studies, and decolonial scholarship. The central aim was to clarify how these bodies of literature connect, assess the evidentiary basis of the proposed pathways, and develop a conceptual framework for community-specific research.

### 2.1 Literature identification and scope

Relevant literature was identified primarily through Scopus and the Web of Science Core Collection. Database searches included records from all publication years available through 2026, allowing foundational scholarship to be considered alongside recent empirical and conceptual work. Searches were conducted across several connected areas corresponding to the main components of the conceptual framework.

Searches combined Indigenous and relevant community-specific terms with concepts related to oral and performative traditions. These included oral tradition, storytelling, oral history, ancestral narrative, myth, legend, song, chant, ritual narrative, proverb, place-based story, and intergenerational transmission. The indexed terms myth and legend were also used because they appear in publication titles, abstracts, and database records. In the analysis itself, community-specific terms were used where available; creation accounts and sacred histories were used when referring more generally to culturally authoritative narratives. Further searches focused on psychological and relational themes, including psychological well-being, mental health, social and emotional well-being, meaning-making, cultural continuity, cultural connectedness, relational selfhood, identity, belonging, collective memory, social cohesion, land, language, spirituality, ancestry, family, and community.

A further group of searches addressed the wider social and institutional context of the study. Relevant terms included modernization, urbanization, formal schooling, market integration, language shift, cultural commodification, epistemic injustice, cultural devaluation, institutional recognition, standardized assessment, and Indigenous knowledge governance.

External valuation emerged through the synthesis of scholarship across these domains. The construct brought together recurring discussions of how Indigenous knowledge, identity, practices, and institutions are assessed through educational, economic, administrative, policy, and cultural criteria.

The reference lists of relevant empirical studies, conceptual articles, and review publications were also examined to identify additional sources, particularly foundational works, community-specific studies, and publications connecting oral traditions with land, language, memory, identity, and intergenerational knowledge.

### 2.2 Selection, exclusion, and treatment of sources

Sources were selected for their contribution to one or more parts of the conceptual argument. The main areas of relevance included Indigenous understandings of psychological or social and emotional well-being; oral, narrative, musical, ritual, historical, and place-based traditions; cultural continuity and cultural connectedness; relational understandings of identity and selfhood; community belonging and participation; and the influence of modernization, institutional valuation, and knowledge marginalization.

Sources were included when they contributed directly to at least one component of the conceptual framework and provided sufficient information about the community, cultural setting, oral practice, or well-being relation under discussion. Sources were excluded when Indigenous peoples or Indigenous knowledge were mentioned only incidentally; when oral tradition was used only as a metaphor or generic label; when the material did not permit the relevant claim to be located in a specific community, practice, or established body of scholarship; or when it consisted of non-scholarly commentary, duplicate reporting, or material without accessible substantive content. Adjacent non-Indigenous scholarship was retained only for conceptual clarification and was not treated as direct evidence concerning Indigenous oral traditions or Indigenous well-being.

The literature included empirical studies, qualitative research, reviews and metasyntheses, theoretical and conceptual articles, scholarly books, and book chapters. Each source was considered according to the claims it could support within the framework. Sources were organized into three evidentiary categories. Community-specific Indigenous studies provided evidence concerning particular peoples, oral practices, and settings. Broader Indigenous health and relational scholarship informed the cultural foundations of the model. Adjacent narrative, ritual, trauma, and oral-tradition scholarship clarified conceptual relations and interpretive possibilities. These categories were retained throughout the synthesis and in Table 1. Community-specific Indigenous studies that directly addressed an oral or performative practice were given priority for claims concerning oral traditions. Broader Indigenous health, well-being, and relational scholarship was used to establish the cultural and psychosocial foundations of the framework. Adjacent narrative, ritual, trauma, and decolonial scholarship was used when it clarified a concept or a possible relation not directly addressed in the available Indigenous literature. Adjacent scholarship did not increase the evidentiary status of a pathway and was not used to generalize findings across Indigenous communities.

**Table 1:** Evidentiary status of the proposed psychosocial pathways.

Proposed pathway	Analytical function	Evidence concerning Indigenous well-being	Evidence specific to oral traditions	Evidentiary status in the model
Meaning-making	An interpretive process through which experience, suffering, memory, and cultural knowledge become socially and culturally intelligible.	Phenomenological and relational research indicates that oral and narrative practices organize time, lived experience, cultural knowledge, and understandings of well-being (Struthers and Peden-McAlpine, 2005; Ullrich et al., 2022).	Community-specific studies of oral tradition, storying, and re-storying illustrate narrative interpretation and relational knowledge exchange in particular settings. These studies clarify how experience is situated within cultural histories and shared systems of meaning.	Context-specific qualitative support for meaning-making as an interpretive process.
Cultural continuity	A temporal and cultural condition connecting past, present, and future through language, memory, cultural practice, identity, and intergenerational transmission.	Metasynthesis and review evidence provide comparatively strong support for the relevance of cultural continuity, cultural connectedness, and broader cultural determinants to Indigenous health and quality of life (Auger, 2016; Brar et al., 2026; Verbunt et al., 2021).	Research on the Kodava *tere* practice provides community-specific evidence of oral tradition as a means of cultural transmission and identity maintenance (Biddappa et al., 2026).	Comparatively stronger empirical support at the broader cultural level, accompanied by community-specific evidence concerning oral transmission.
Relational selfhood	A mode of personhood and self-understanding grounded in relations with land, ancestry, language, spirituality, family, community, and collective memory.	Empirical studies support the importance of land, cultural reconnection, family, community, intergenerational continuity, and self-determination for Indigenous identity and well-being (Linjean and Semanchin Jones, 2025; Salusky et al., 2022; Smutz et al., 2025; Ullrich et al., 2025; Wexler, 2014).	Community-specific studies relate oral or story-based practices to identity, belonging, cultural transmission, resistance, and cultural reclamation (Beals et al., 2025; Biddappa et al., 2026). These studies provide context-specific evidence concerning related forms of identity and belonging, but they do not examine relational selfhood as a specified psychological construct or isolate the contribution of oral traditions.	Broader indirect empirical support for relational foundations, accompanied by limited context-specific evidence concerning oral and story-based practices. The proposed relation between oral traditions and relational selfhood remains open to direct empirical examination.
Social cohesion	A community-relational process involving collective belonging, participation, recognition, reciprocity, mutual support, intergenerational exchange, and the negotiation of authority and difference.	Qualitative research associates relational knowledge exchange, cultural narratives, collective participation, identity, and cultural transmission with community connection and Indigenous well-being (Fernandez and Beltrán, 2022; Ullrich et al., 2022).	Studies of cultural dance narratives and the Kodava *tere* practice show how narrative and performative practices are embedded in collective participation, identity, and transmission (Fernandez and Beltrán, 2022; Biddappa et al., 2026).	Context-specific qualitative support concerning collective belonging, participation, and cultural transmission.

The classifications were determined from three criteria: direct engagement with oral traditions, specificity of the Indigenous or community context, and the type of support offered for the proposed relation. The table separates analytical function from evidentiary strength and summarizes the literature included in the present conceptual analysis.

Studies examining particular oral, narrative, musical, ritual, or story-based practices in identifiable Indigenous settings provided context-specific evidence concerning those practices. Broader research on Indigenous land relations, language, cultural reconnection, family, community, identity, self-determination, and well-being informed the cultural and relational foundations of the model. Scholarship on oral knowledge, narrative, trauma, epistemic injustice, and decolonial psychology contributed to the interpretation of concepts and relations where direct Indigenous oral-tradition research remained limited.

Attention was also given to the cultural and geographical setting of each study, the oral form under discussion, and the conditions under which knowledge was transmitted or shared. Storytelling, songs, oral histories, ritual narratives, proverbs, and place-based accounts were therefore considered in relation to their particular community and context. This approach preserved the specificity of the original studies while allowing recurring conceptual relations to be compared across the literature.

### 2.3 Conceptual synthesis and evaluation

The synthesis developed through an iterative comparison of the literature. Sources were first organized around the main areas of the study: modernization, institutional change, and external valuation, Indigenous oral traditions and knowledge systems, culturally grounded well-being, relational identity, and community belonging. Within these areas, attention was given to recurring relations involving narrative interpretation, intergenerational transmission, land and language, cultural connection, shared participation, and collective memory.

The comparison produced four pathways with distinct analytical functions. Meaning-making captures interpretive processes through which experience and suffering become culturally intelligible. Cultural continuity addresses the transmission and renewal of memory, language, knowledge, values, and identity across generations. Relational selfhood concerns forms of personhood and self-understanding grounded in land, ancestors, family, spirituality, language, and community. Social cohesion refers here to collective belonging and participation through shared narration, recognition, reciprocity, mutual support, and intergenerational exchange.

The evidentiary basis of each pathway was evaluated through three criteria: direct engagement with oral traditions, specificity of the Indigenous or community context, and the type of support offered for the proposed relation. Broader empirical research established the relevance of cultural continuity, connectedness, land, family, community, and relational identity to Indigenous well-being. Community-specific studies illustrated how particular oral, narrative, musical, ritual, or performative practices operated in identifiable settings. Adjacent scholarship contributed conceptual clarification where direct oral-tradition evidence remained limited.

Each publication used in the pathway analysis was reviewed in relation to the people or community studied, geographical and historical setting, oral form, study design, and claim relevant to the framework. Claims in the main text and Table 1 were then compared with these source notes. Community-specific findings were retained at the level of the community and practice examined unless a broader relation was also supported by Indigenous scholarship from other settings. Sources that qualified or limited a proposed relation were retained in the synthesis. Where the literature supported only a context-bound illustration or conceptual possibility, the wording remained at that level.

These distinctions guided the interpretation of the four pathways and the classifications presented in Table 1. The pathways are treated as interrelated and context-dependent, with each source interpreted in relation to the people, setting, language, historical conditions, and oral practice it addresses. Their relevance is shaped by community history, language vitality, land relations, access to knowledge holders, patterns of disruption and renewal, and local authority over transmission and use. Community authority, cultural protocols, knowledge governance, and conditions of access are therefore treated as integral to the interpretation and application of the framework.

## 3 Modernization, external valuation, and indigenous psychological well-being

Modernization is used here to describe a cluster of overlapping institutional processes that reorganize the conditions under which Indigenous knowledge, identity, language, labor, and cultural practices are recognized. These processes include urbanization, market incorporation, industrial and extractive development, formal schooling, state administration, language shift, cultural commodification, and digital circulation. This usage emphasizes institutional reorganization and changing evaluative conditions rather than a linear transition from tradition to modernity. Indigenous communities actively negotiate these processes through adaptation, refusal, resurgence, governance, language renewal, cultural innovation, and the creation of contemporary futures.

This article uses external valuation as a middle-range analytical construct for examining how Indigenous knowledge, practices, identities, and institutions are assessed through criteria established outside the communities concerned. The construct has three defining features: the operative standards of worth originate primarily in external institutions; these standards influence recognition, legitimacy, resources, or public visibility; and community-defined relational, cultural, spiritual, or place-based values receive reduced institutional weight. External valuation operates across educational, administrative, economic, health, environmental, and cultural domains through practices such as standardized assessment, resource appraisal, policy recognition, market valuation, and cultural commodification (Damoah et al., 2024; Graben, 2013; Kearney, 2021; Lewis et al., 2025; Miller, 2018; Neill et al., 2026). Coloniality, epistemic injustice, structural racism, and misrecognition describe wider structures or relations; external valuation isolates the evaluative act through which institutional standards assign legitimacy and worth. Its analytical contribution lies in connecting broader power relations with recurring acts of assessment across institutional settings. Community-controlled evaluation and negotiated recognition remain grounded in locally authorized criteria and therefore sit outside the construct.

One important site of external valuation is the changing status of Indigenous and local knowledge systems. Research on local ecological knowledge has shown that such knowledge systems are increasingly threatened by market integration, cultural homogenization, environmental change, and weakening intergenerational transmission (Aswani et al., 2018; Fernández-Llamazares et al., 2021). These threats are not only technical losses of information about plants, animals, land, or ecological management. They also affect the cultural worlds in which knowledge is learned, practiced, and valued. When Indigenous knowledge is displaced by externally validated forms of expertise, the community does not simply lose a set of practices. It may also lose part of the symbolic and relational structure through which people understand responsibility, memory, place, and belonging.

Language loss reveals a similar dynamic. Indigenous languages are often weakened by schooling, state institutions, migration, and everyday pressures to use dominant languages. Yet language is not only a communicative tool. It carries relational meanings, spiritual orientations, place-based memory, and intergenerational knowledge. Studies on Indigenous language loss and revitalization suggest that language reclamation can be tied to cultural practice, community pride, identity, and well-being (Jennings, 2022; Khawaja, 2021; Sivak et al., 2019). The psychosocial significance of language loss therefore lies not only in linguistic decline, but in the weakening of a medium through which people inherit categories of relation, memory, and self-understanding.

Economic transformation also reorganizes Indigenous value. Market integration may create new opportunities, but it can also pressure Indigenous communities to translate their ways of life into economic terms. Research on Indigenous economies in Arctic regions, for example, describes the tension between tradition, market, and state in the transformation of Indigenous economic activities (Gladun et al., 2022). Likewise, research on Indigenous entrepreneurs in Northern Australia suggests that success cannot be fully understood through non-Indigenous business metrics alone, because Indigenous entrepreneurial goals may include cultural preservation, community responsibility, and non-financial forms of value (Austin and Garnett, 2018). These studies show why external valuation is psychologically important. If success is defined only by market expansion, income, productivity, or formal business growth, then culturally grounded forms of achievement may become invisible or undervalued.

Education is another domain in which Indigenous lives are often measured by external standards. Standardized assessment and dominant schooling practices may present themselves as neutral, yet they frequently privilege particular forms of knowledge, communication, reasoning, and achievement. Work on culturally responsive assessment for Indigenous students has challenged the adequacy of assessment systems that fail to reflect Indigenous cultural and linguistic contexts (Trumbull and Nelson-Barber, 2019). Schmelkes (2018) similarly argues for an intercultural approach to evaluation, while Huaman (2022) shows how Indigenous scholarship can reshape the field of comparative education through pluriversal and decolonial orientations. These discussions matter for psychological well-being because education is not only a system for measuring learning. It is also a system through which young people learn whose knowledge counts, whose language is legitimate, and whose future is imaginable.

The psychosocial effects of external valuation emerge through specific historical and institutional conditions. Literature on structural racism, historical trauma, and Indigenous mental health associates the devaluation of culture, knowledge, language, and identity with alienation, shame, cultural insecurity, marginalization, and weakened belonging (González et al., 2022; Juutilainen et al., 2014; Nutton and Fast, 2015). These experiences arise within social contexts where Indigenous histories and ways of knowing have been suppressed, misrecognized, or required to justify themselves before dominant institutions.

Historical and collective trauma further complicate any purely individual account of psychological well-being. Studies of residential and boarding school experiences, substance use, and intergenerational harm show that Indigenous distress is often linked to long histories of cultural suppression and structural violence rather than to isolated individual pathology (Juutilainen et al., 2014; Nutton and Fast, 2015; O’Loughlin, 2009). This does not mean that all Indigenous suffering can be reduced to colonial history, nor that Indigenous communities should be represented only through trauma. It does mean that psychological well-being cannot be separated from the conditions under which culture, language, land, and community relations have been disrupted.

Indigenous communities also shape contemporary conditions through refusal, resurgence, language renewal, political mobilization, self-determined governance, cultural innovation, and selective engagement with urban and digital forms. Language revitalization, Indigenous education, and cultural practice renew relations among people, place, memory, and identity (Jennings, 2022; Khawaja, 2021; Sivak et al., 2019). Oral traditions participate in this agency by carrying historical memory, renewing political and cultural claims, and supporting the creation of contemporary Indigenous futures. Their significance therefore includes cultural continuity, active world-making, and the assertion of community authority.

This section therefore positions modernization as a psychosocial problem rather than only a socioeconomic condition. Its significance lies not simply in urban migration, industrial labor, market participation, or schooling as such, but in the evaluative order that accompanies them. When Indigenous knowledge is recognized only as heritage, when culture is valued mainly as tourism or commodity, when language is treated as secondary to dominant communication systems, and when success is defined through external metrics, Indigenous identity may become vulnerable to misrecognition. Such misrecognition can affect how people understand themselves, their communities, and their place in the world.

The following sections examine oral traditions as culturally situated practices through which meaning, continuity, relational self-understanding, and collective belonging are sustained and renewed under changing institutional conditions.

## 4 Decolonial perspectives on indigenous knowledge

The argument of this article requires a decolonial perspective because the marginalization of Indigenous oral traditions involves continuing hierarchies of knowledge and authority. Dominant psychological, public health, and social science traditions often privilege written documentation, formal theory, measurable evidence, and institutional expertise. Indigenous oral traditions carry their own epistemic, relational, ethical, and psychosocial functions through community-held systems of interpretation and authority. A decolonial perspective directs attention to who defines knowledge, whose forms of knowing receive institutional recognition, and how these relations shape well-being.

Decolonial critiques of psychology are especially relevant here because they question the assumption that psychological science can stand outside history and culture. Rather than viewing dominant psychology as a neutral universal framework, decolonial scholarship draws attention to the ways psychological knowledge may reproduce hegemonic assumptions about selfhood, development, rationality, health, and evidence (Adams et al., 2017, 2018). This critique does not require rejecting psychology as a field. It requires asking how psychology can engage Indigenous knowledge without absorbing it into pre-existing categories that were not built from Indigenous lifeworlds. For the present article, this means that oral traditions should not be approached only as culturally interesting examples of narrative, coping, or communication. They should be examined as knowledge practices with their own social authority, interpretive rules, and relations to community life.

This is why the distinction between oral tradition as folklore and oral tradition as knowledge is crucial. Work on oral traditions shows that they are not simply informal stories that survive before literacy or outside formal education. They can be complex systems for preserving, interpreting, and transmitting knowledge across generations. Ikuenobe (2018), for instance, discusses oral tradition in relation to epistemic dependence and knowledge in African cultures, while Foley (2013) describes oral traditions through a comparative lens that emphasizes their communicative and interpretive complexity. Afolayan (2021) also treats folklore and oral traditions as pedagogical media, and Marks (2010) stresses the autonomous world of oral legend. Taken together, these studies support a key point for this article: oral traditions are not static remnants of the past. They are living knowledge systems that require contextual fluency, shared memory, and culturally situated interpretation.

This point has direct implications for psychological well-being. As knowledge systems, oral traditions become relevant to well-being through the ways they organize meaning, identity, moral orientation, social memory, and belonging. To call them “knowledge” is therefore not simply to elevate their status in academic terms. It is to recognize that they help structure the worlds in which people understand suffering, responsibility, continuity, and repair. When such knowledge is dismissed as myth or folklore in the reductive sense, Indigenous communities may not only lose cultural recognition; they may also be deprived of legitimate frameworks through which well-being is understood and sustained.

The concept of epistemic injustice helps clarify this problem. In mental health research and practice, epistemic injustice occurs when some people’s knowledge, testimony, or lived experience is treated as less credible because it does not fit dominant assumptions about expertise. Work on epistemic injustice in mental health research emphasizes the need to take lived experience seriously, while recent discussion of Indigenous mental healthcare in Bangladesh links epistemic injustice to the marginalization of Indigenous perspectives in global mental health (Okoroji et al., 2023; Omar Faruk, 2025). More broadly, Cummings et al. (2023) show how epistemic injustice can be applied to real-world development contexts. These discussions are useful for the present argument because they show that knowledge exclusion is not abstract. It can shape which forms of suffering are recognized, which forms of healing are considered legitimate, and which communities are allowed to define their own well-being.

A decolonial approach also requires attention to knowledge sovereignty. Indigenous communities are not merely sources of cultural data for researchers, clinicians, or policy makers. They are knowledge-holding communities with their own protocols, authorities, and responsibilities. In Indigenous research, this has often been expressed through attention to relationship-building, community participation, and sustainable intervention outcomes. Chilisa and Tsheko (2014), for example, emphasize the importance of building relationships in Indigenous mixed methods research. Baur (2021) similarly foregrounds positionality in decolonizing social science methodology. Such work reminds us that research on Indigenous oral traditions cannot be ethically separated from the social relations through which those traditions are held, transmitted, and authorized.

This matters particularly for mental health interventions. There is a risk that Indigenous stories, rituals, songs, or cultural practices may be extracted from their communities and repackaged as therapeutic resources without adequate attention to context, authority, or protocol. Such extraction may appear respectful on the surface because it includes “culture,” but it can still reproduce a colonial relation if Indigenous knowledge is used without Indigenous control over meaning, access, and application. In this sense, cultural inclusion is not the same as cultural safety. Cultural inclusion may add Indigenous elements to an existing intervention, while cultural safety asks whether the intervention respects the community’s own knowledge systems, relational obligations, and conditions of use.

This distinction is important for the conceptual model developed in this article. Oral traditions are not proposed here as techniques that can be removed from Indigenous contexts and inserted into generic psychological programs. They are understood as living psychosocial knowledge embedded in language, land, memory, kinship, spirituality, and community authority. Their possible contribution to well-being depends precisely on these relations. If they are detached from them, their meaning may change, weaken, or become appropriated by external institutions. A decolonial framework therefore prevents the argument from becoming a simple celebration of storytelling as therapy. It asks how storytelling, creation accounts and sacred histories, songs, and ritual narratives remain accountable to the communities from which they emerge.

This theoretical position clarifies why Indigenous oral traditions should be treated as legitimate objects of psychological analysis rather than as marginal cultural illustrations. It also sets ethical limits on how their psychosocial significance can be discussed. Oral traditions illuminate forms of well-being that mainstream psychology may overlook, including relational selfhood, cultural continuity, collective memory, and socially grounded meaning-making. A decolonial perspective is therefore not an added theoretical decoration. It is the basis for asking how Indigenous knowledge can be engaged without being subordinated to the very systems of valuation that have historically marginalized it. With this epistemic and ethical position established, the next section examines the psychosocial significance of oral traditions in greater detail.

## 5 Indigenous oral traditions as living psychosocial knowledge

Indigenous oral traditions are conceptualized in this article as living psychosocial knowledge embedded in community-held epistemic and relational infrastructures. Psychosocial refers to the organization of personal experience through relational, cultural, historical, spiritual, ethical, and community conditions. Living psychosocial knowledge is the analytical term used in this synthesis for oral practices that actively organize and transmit meaning, memory, identity, ethical orientation, and relational belonging across generations. As epistemic infrastructures, oral traditions organize, interpret, and transmit knowledge concerning history, ethics, spirituality, land, identity, and community life. As relational infrastructures, they sustain connections among persons, generations, ancestors, language, place, and collective memory.

The category of oral tradition is broad. It may include storytelling, creation accounts and sacred histories, legends, songs, chants, ritual speech, prayers, ancestral narratives, place-based accounts, oral histories, proverbs, and intergenerational narration. Creation accounts and sacred histories are understood here as culturally authoritative forms of knowledge and memory whose meanings are grounded in the communities that hold and transmit them. Where the term myth appears as a source term or as a reductive label under critique, it does not classify culturally authoritative narratives as false or fictional. These forms should not be collapsed into a single genre. Songs may carry historical memory differently from creation accounts and sacred histories; ritual narratives may organize spiritual or ethical relations differently from family oral histories; place-based stories may connect memory to landscape in ways that cannot be reduced to general cultural identity. Still, across these diverse forms, oral traditions often work as more than expressive culture. They transmit knowledge, organize memory, and shape how persons and communities understand their place in the world (Caesar and Sanasam, 2018; Fausto et al., 2011; Frey, 2007; Mahuika, 2021; Mukuni, 2025). The available literature also cautions against treating these forms as interchangeable units. Existing studies generally examine particular practices in particular cultural settings rather than comparing oral forms across Indigenous communities. For example, the Kodava tere practice has been examined as a cultural conveyor, Indigenous storytelling has been discussed in relation to ecological knowledge and conservation, and research in Namibia has addressed the digitization of Indigenous storytelling (Jose and Rahul, 2026; Biddappa et al., 2026; Rodil and Winschiers-Theophilus, 2016). These studies do not establish systematic differences in authority, access restrictions, or psychosocial effects. They do, however, support a more limited conclusion: claims about the functions of oral traditions should be tied to the specific form, community, setting, and conditions of transmission under examination.

One central function of oral traditions is memory. This does not refer only to the retention of past events. Oral traditions can preserve collective histories, ancestral relations, ecological knowledge, migration memories, moral lessons, and experiences of survival. Studies of Indigenous oral histories show that oral accounts may construct historical consciousness in ways that are not dependent on written archives alone (Crowell and Howell, 2013; Khiangte et al., 2025; Mahuika, 2021). In this sense, oral traditions do not merely supplement written history. They may offer their own temporal logic, in which the past remains active in the present and continues to shape future obligations. Struthers and Peden-McAlpine (2005) similarly suggest that oral tradition is important for understanding time and experience in Indigenous research contexts. Such work supports the view that oral traditions carry not only information, but a way of inhabiting time.

This temporal dimension is especially important for psychological well-being. A person who receives oral tradition does not simply learn facts about the past. They may also receive a location within a chain of relations. Stories can tell people who they are, where they come from, how they are connected to land and ancestors, and what responsibilities follow from these connections. Oral traditions may connect personal identity with collective memory. Kukutai et al. (2020), in their discussion of survivance as narrative identity, show how oral history can be tied to the ongoing assertion of Indigenous presence and identity. This is not a passive form of remembrance. It is a way of narrating survival, continuity, and self-understanding in the face of historical disruption.

Oral traditions also have pedagogical and ethical functions. They teach values, responsibilities, and forms of conduct, but they do so in ways that are often embedded in story, performance, song, ritual, or embodied practice. Proverbs, for example, may carry ethical and ecological lessons; ritual speech may teach relations between human beings, spiritual forces, and place; songs may preserve historical knowledge while also sustaining communal participation (Anwar, 2020; Dais-Mohadali, 2025; Fausto et al., 2011; Progress and Peter, 2026). This mode of teaching is not secondary to formal education. It reflects a different organization of knowledge, one in which ethics, memory, affect, and social belonging are learned together.

The emotional and spiritual dimensions of oral traditions are also evident in the culturally situated ways that stories, songs, chants, and ritual narratives hold experiences of grief, fear, displacement, conflict, and cultural loss. They can also sustain spiritual connection and cultural continuity, particularly when they are performed within community settings or transmitted through elders, healers, ritual specialists, families, and other knowledge holders. Work on cultural continuity, healing voices, verbal arts, and music suggests that oral forms can connect emotional experience to shared cultural meaning (Fausto et al., 2011; Neumann, 2025). Their psychological significance lies not in functioning as clinical treatment in a narrow sense, but in providing culturally meaningful structures through which emotion and relation can be held.

Oral traditions are also place-making practices. Many Indigenous narratives are tied to specific lands, waters, routes, settlements, ecological features, or sacred places. Such stories do not simply describe landscape. They make place intelligible as memory, relation, and responsibility. Work on oral tradition and archeology in southeastern Alaska, for example, shows how oral accounts can connect historical memory, place, and material evidence (Crowell and Howell, 2013). Environmental and literary studies of Indigenous storytelling likewise suggest that stories may connect people and land in ways that challenge abstract or purely physical understandings of environment (Anwar, 2020; Korteweg et al., 2010). This matters for psychological well-being because belonging is not only a social feeling. In many Indigenous contexts, belonging is also place-based and storied.

The concept of living knowledge is useful because it avoids two misunderstandings. The first is the idea that oral traditions are fragile survivals from a disappearing past. The second is the opposite romantic idea that oral traditions remain unchanged across time. Oral traditions endure because they are transmitted, reinterpreted, and renewed. They can preserve core values while also responding to new social, political, ecological, and intergenerational conditions. Coker (2021) discusses oral traditions and folklore in terms of retrospect and future prospects, while Mahuika (2021) rethinks oral history and tradition from an Indigenous perspective. Hundleby (2025), in the context of Solomon Islands song, also shows how song can involve preservation and transformation at the same time. These works support a more dynamic view: oral traditions are not static heritage, but practices through which continuity and change are negotiated.

This dynamic character is central to their psychosocial significance. If oral traditions only preserved the past, their relevance to contemporary well-being would be limited to cultural memory. But if they are living knowledge, they can help communities interpret contemporary pressures, including modernization, displacement, cultural commodification, language loss, and external valuation. They may provide narrative resources for understanding disruption without accepting the idea that Indigenous identity is merely damaged or obsolete. They may also offer ways of imagining continuity without denying change. In this respect, oral traditions can connect past, present, and future not as a simple linear sequence, but as a relational field in which ancestral memory, present struggle, and future responsibility are held together.

For this article, the phrase “living psychosocial knowledge” therefore names the intersection of several functions. Oral traditions are “living” because they are performed, transmitted, adapted, and reinterpreted across generations. They are “psychosocial” because they shape meaning, identity, emotional expression, social cohesion, and belonging. They are “knowledge” because they encode and transmit understandings of history, ethics, spirituality, land, and community. This formulation locates their psychological relevance in the relational worlds they sustain.

## 6 Psychosocial pathways: from external valuation to relational selfhood

The previous section established the epistemic and relational role of Indigenous oral traditions. This section identifies four pathways through which such knowledge becomes relevant to culturally grounded psychological well-being under external systems of valuation.

Four pathways are proposed: meaning-making, cultural continuity, relational selfhood, and social cohesion. Pathway serves here as an organizing term for four different analytical functions. Meaning-making describes an interpretive process; cultural continuity describes a temporal and cultural condition; relational selfhood describes a mode of personhood and self-understanding; and social cohesion describes relational practices of collective belonging and participation. Their interaction is recursive, and a single oral practice may engage several pathways at once. The relevance of each pathway is shaped by the particular practice, community, language, setting, cultural authority, and conditions of transmission involved. Section 6.6 evaluates the different forms and strengths of evidence supporting each pathway.

### 6.1 Meaning-making: narrating suffering beyond individual failure

One psychosocial function of oral traditions is meaning-making. In contexts shaped by displacement, historical trauma, cultural suppression, and marginalization, psychological suffering may easily be interpreted through individualized terms: personal weakness, failure to adapt, emotional dysfunction, or lack of resilience. Oral traditions can offer another interpretive frame. Through storytelling, ritual narratives, creation accounts and sacred histories, songs, and collective narration, experiences of grief, loss, trauma, and disruption may be placed within broader histories of community survival, cultural continuity, and collective struggle.

This does not mean that oral traditions erase suffering or provide simple consolation. Their significance lies in the way they can make distress narratable. Work on grief and trauma in Aboriginal communities in Canada has emphasized that healing cannot be separated from community, cultural understanding, and collective experience (Poonwassie, 2006). Studies of storytelling and ritual in post-conflict settings and adjacent narrative scholarship also suggest that narrative and ritual practices may help transform painful experience into shared meaning, social recognition, and possibilities for repair (Beltrán and Begun, 2014; Fuertes, 2012; Kamwaria, 2022). These studies do not justify a generalized claim that all storytelling is healing. Where adjacent narrative or post-conflict studies are cited, they are used to clarify conceptual possibilities rather than as direct evidence concerning Indigenous oral traditions. They do support a more careful claim: narrative and ritual practices can help communities organize suffering in ways that are socially and culturally intelligible.

For Indigenous psychological well-being, this is important because distress is often not only intrapsychic. It may be tied to land loss, language suppression, forced schooling, racism, cultural devaluation, and intergenerational trauma. Oral traditions can help shift the interpretation of suffering from isolated personal failure to relational and historical experience. A person’s grief or insecurity may then be understood as part of a wider pattern of cultural disruption, while also being held within narratives of endurance and renewal. This movement from isolated distress to shared meaning is one reason oral traditions can be considered psychosocial rather than merely expressive.

### 6.2 Cultural continuity: holding identity across time

A second pathway is cultural continuity. Cultural continuity refers to the felt and practiced connection between past, present, and future. It is sustained through language, ceremonies, land-based knowledge, family memory, traditional practices, and community institutions. In the context of this article, oral traditions are central to cultural continuity because they transmit more than information. They carry ways of remembering, valuing, relating, and imagining the future.

The link between cultural continuity and well-being has been developed in several areas of Indigenous health research. Auger’s (2016) metasynthesis identifies cultural continuity as an important determinant of Indigenous peoples’ health, while research on cultural identity and social and emotional well-being among Aboriginal and Torres Strait Islander children suggests that cultural identity can be connected to better social–emotional outcomes (Fatima et al., 2022). Work on cultural connectedness and health-related quality of life across CANZUS countries, namely Canada, Australia, New Zealand, and the United States, similarly supports the broader relevance of cultural ties to health and well-being (Brar et al., 2026). These studies indicate that culture should not be treated as a decorative or secondary dimension of well-being. Cultural continuity can be part of the structure through which people develop identity security, purpose, and resilience.

Oral traditions contribute to this continuity by keeping ancestral memory and community knowledge active. Elders, storytellers, healers, families, and community gatherings often serve as sites of transmission, allowing younger generations to inherit stories, songs, values, and place-based meanings (Mahuika, 2021; Mukuni, 2025; Neumann, 2025). This inheritance is not simply a return to the past. It gives the present a lineage and gives the future a direction. For Indigenous youth in particular, the ability to perceive oneself across time may be shaped by community and cultural factors (Klassen et al., 2024). Oral traditions can therefore support self-continuity: the sense that one’s present self is connected to those who came before and to those who will come after.

This temporal dimension matters under modernization because external valuation may contribute to conditions of cultural discontinuity. When dominant institutions treat Indigenous languages as secondary, traditional practices as outdated, or Indigenous knowledge as symbolic rather than authoritative, younger generations may experience identity uncertainty or cultural distance. Oral traditions may respond to this condition by supporting or renewing continuity. They remind individuals that their value is not limited to present external recognition. It is also grounded in ancestral relations, cultural memory, and collective survival.

### 6.3 Relational selfhood: regrounding self-worth in land, ancestors, and community

The third pathway is relational selfhood. In this framework, relational selfhood refers to forms of personhood and self-understanding grounded in relations with land, ancestors, language, spirituality, family, community, and collective memory. The term is used comparatively across the reviewed literature while retaining the meanings attached to each people, community, and knowledge system. Its form is shaped by local ontologies, histories, responsibilities, and relations of authority.

This pathway directly responds to the problem of external valuation discussed earlier. Under external valuation, Indigenous persons and communities may be judged through standards such as productivity, educational achievement, market success, development, formal recognition, or cultural commodification. These standards may orient self-evaluation toward dominant criteria, making self-worth increasingly dependent on conformity with prevailing systems of value. Oral traditions can support a different orientation. By narrating relations to land, ancestors, sacred places, community responsibilities, and cultural memory, they may provide relational grounds for self-understanding and self-worth beyond external measurement.

Several bodies of literature support this conceptual shift. Work on land, language, and sustainability emphasizes that Indigenous relations to land and language are often mutually constitutive, not merely symbolic (Ferguson and Weaselboy, 2020). Studies of ancestral homelands and sacred landscapes also indicate that land can be bound to culture, identity, law, and spiritual responsibility (Assumi, 2026; LaDuke, 2017; Smutz et al., 2025). In this view, land is not only territory, resource, or environment. It is part of the relational structure through which selfhood and belonging are formed. Oral traditions carry these relations by telling people how places came to matter, how obligations are inherited, and how human life is situated within broader ecological and spiritual worlds.

Relational selfhood is therefore not only an abstract philosophical concept. It is proposed here as psychosocially relevant to how self-worth, belonging, and resilience are understood. If self-worth is derived only from external recognition, then exclusion, marginalization, and cultural devaluation may produce shame or insecurity. If self-worth is also grounded in land, ancestors, language, and community memory, then the self has other sources of value from which to draw. This is not a guarantee against distress, but it may provide a culturally grounded basis for resilience. The movement from external valuation to relational selfhood is thus one of the central conceptual contributions of this article.

### 6.4 Social cohesion: collective belonging and participation through shared narration

In this framework, social cohesion refers to collective belonging and participation expressed through shared narration, recognition, reciprocity, mutual support, and intergenerational exchange. It also includes the negotiation of authority, memory, gender relations, generational responsibilities, and cultural change within communities. Oral traditions may contribute to these relational processes when they are shared, performed, remembered, and renewed in ceremonies, family gatherings, healing practices, educational spaces, and community events.

The relation between cultural participation and well-being has been noted in both rural and urban Indigenous contexts. Hossain and Lamb (2020) examine cultural attachment and well-being among Indigenous people in Canada, while Firestone et al. (2024) discuss mental health and cultural continuity among an urban Indigenous population in Toronto. These studies are important because they show that Indigenous cultural connection is not relevant only in remote or rural settings. Even under urban conditions, cultural continuity and attachment may remain significant for well-being. Oral traditions can be one means through which such connection is sustained.

Social cohesion is also supported by the roles of elders, storytellers, healers, and knowledge holders. Their significance is not only that they possess information. They help maintain the relationships through which knowledge remains meaningful. Work on oral history and tradition from Indigenous perspectives emphasizes the importance of intergenerational transmission, while studies of curanderismo and cultural continuity show how healing voices and cultural practices can sustain identity and community connection (Mahuika, 2021; Neumann, 2025). Similarly, research on oral tradition as a cultural conveyor suggests that oral practice can function as a medium of collective identity and social continuity (Biddappa et al., 2026). In these cases, oral traditions do not merely communicate content. They gather people into shared memory.

This gathering function matters psychologically. Alienation, shame, identity insecurity, and cultural disconnection often intensify when people feel alone with their experiences. Shared stories, songs, rituals, and oral histories may counter this isolation by placing individual experience within a community of listeners, speakers, ancestors, and future generations. Belonging is therefore not only an emotional state. It may be sustained through repeated practices of narration, listening, recognition, and participation.

### 6.5 Integrating the pathways

These four pathways—meaning-making, cultural continuity, relational selfhood, and social cohesion—specify the conceptual relations through which Indigenous oral traditions may become relevant to psychological well-being. Meaning-making allows suffering to be interpreted within collective histories rather than only individual failure. Cultural continuity links identity across generations. Relational selfhood grounds self-worth in land, ancestors, language, spirituality, and community rather than external standards alone. Social cohesion may sustain belonging through shared narration and communal participation.

The pathways also clarify why oral traditions remain significant under modernization. Modernization and external valuation may pressure Indigenous communities to translate their knowledge, labor, identity, and culture into dominant standards of success. Oral traditions offer a different ground of meaning. They do not simply preserve the past; they help sustain the relational conditions through which people can understand who they are, where they belong, and why their cultural worlds remain valuable. Together, the four pathways clarify how oral traditions may become relevant to culturally grounded psychological well-being.

These pathways should be understood through local histories, languages, protocols, authorities, and social conditions. Oral traditions may be differently available, differently valued, and differently renewed across Indigenous communities. The framework proposed here is therefore most useful as a conceptual guide for community-led and context-specific research on how oral traditions become relevant to well-being in particular Indigenous settings.

### 6.6 Evidentiary status of the proposed pathways

The four pathways do not have equivalent evidentiary status. Cultural continuity has the strongest support in the broader Indigenous health and well-being literature. A metasynthesis of qualitative research identifies cultural continuity as an important determinant of Indigenous health, while review evidence also links cultural connectedness and broader cultural determinants with health and quality of life (Auger, 2016; Brar et al., 2026; Verbunt et al., 2021). These studies support the relevance of cultural continuity, but they generally examine culture and connectedness as broad domains rather than isolating oral traditions. Research on the Kodava tere practice offers more specific but context-bound support for oral tradition as a means of cultural transmission, without establishing a general causal relation between oral tradition and well-being (Biddappa et al., 2026).

Meaning-making and social cohesion have more limited and context-specific support. Phenomenological work has examined oral tradition in relation to time and lived experience, while research on storying and re-storying has connected narrative practice with relational knowledge exchange and Indigenous well-being (Struthers and Peden-McAlpine, 2005; Ullrich et al., 2022). Research on Indigenous cultural dance narratives and the Kodava tere practice further suggests that culturally situated narrative and performative practices may be embedded in collective participation, identity, and transmission (Fernandez and Beltrán, 2022; Biddappa et al., 2026). These studies support the plausibility of meaning-making and social connection in particular settings, but they do not provide comparative or causal tests of these pathways.

Relational selfhood has a mixed evidentiary basis. A small number of context-specific studies connect oral or story-based practices with identity, belonging, cultural transmission, resistance, or cultural reclamation (Beals et al., 2025; Biddappa et al., 2026). These studies provide partial support for the proposed pathway, but they do not test relational selfhood as a clearly specified psychological construct or establish oral tradition as its cause.

A broader body of empirical research supports relational understandings of Indigenous identity through land, cultural reconnection, family, community, intergenerational continuity, and self-determination (Linjean and Semanchin Jones, 2025; Salusky et al., 2022; Smutz et al., 2025; Ullrich et al., 2025; Wexler, 2014). Because these studies do not isolate the specific contribution of oral traditions, they provide indirect rather than direct support for the proposed pathway. The specific contribution of oral traditions to relational selfhood therefore remains open to community-specific empirical investigation.

The model consequently distinguishes among three forms of support: broader empirical evidence concerning cultural continuity and the relational conditions of Indigenous well-being; limited and context-specific evidence involving particular oral, narrative, or performative practices; and theoretically informed propositions whose specific causal processes remain untested. None of the four pathways should be interpreted as a universal causal relation or as operating in the same manner across Indigenous communities. Evidentiary status should also be distinguished from analytical role. A construct may be empirically associated with well-being without functioning as an indicator of well-being in the present model. The four concepts are retained as proposed pathways, while their analytical functions remain distinct. Meaning-making and social cohesion concern processes, cultural continuity is a temporal and cultural condition, and relational selfhood is a mode of personhood and self-understanding. Identity security, belonging, self-continuity, and cultural connectedness remain indicators of the culturally grounded well-being outcome. These distinctions provide the evidentiary basis for the conceptual model presented in the next section.

## 7 A conceptual model of oral traditions and indigenous psychological well-being

Drawing together the preceding sections, this article proposes a conceptual model connecting modernization pressures, external valuation, psychosocial risks, Indigenous oral traditions, four psychosocial pathways, and culturally grounded psychological well-being. The model organizes these elements within a relational framework and identifies their connections as subjects for community-specific empirical examination.

The first component of the model is modernization pressure. In this model, modernization refers to the overlapping institutional processes discussed in Section 3, including urbanization, market incorporation, industrial and extractive development, formal schooling, state administration, language shift, cultural commodification, and digital circulation. These processes reorganize the conditions under which Indigenous knowledge, identity, language, and cultural practices receive recognition and institutional value. Research on Indigenous and local knowledge systems shows that market incorporation, cultural homogenization, and weakened intergenerational transmission can place pressure on knowledge systems and community continuity (Aswani et al., 2018; Fernández-Llamazares et al., 2021). At the same time, Indigenous communities engage these conditions through adaptation, language renewal, cultural resurgence, self-determined governance, and cultural innovation (Austin and Garnett, 2018; Sivak et al., 2019; Trumbull and Nelson-Barber, 2019).

The second component is external valuation. It identifies the evaluative act through which externally established institutional standards assign legitimacy and worth to Indigenous knowledge, practices, identities, and institutions. It is analytically narrower than coloniality or structural racism and broader than any single domain-specific practice, such as standardized educational assessment, resource appraisal, health resource allocation, or cultural commodification. The construct allows these evaluative practices to be compared across institutional domains without assuming that they operate identically or produce uniform psychosocial consequences (Damoah et al., 2024; Graben, 2013; Kearney, 2021; Lewis et al., 2025; Miller, 2018; Neill et al., 2026). Modernization pressures provide contemporary institutional settings in which longer colonial and evaluative orders are reproduced, reorganized, and intensified.

The third component is psychosocial risk. External valuation can contribute to alienation, shame, identity insecurity, cultural disconnection, weakened belonging, and the loss of self-continuity. These risks should not be interpreted as individual pathology detached from history. They are social and psychological at the same time. Studies of historical trauma, cultural continuity, and Indigenous mental health suggest that distress is often shaped by collective histories of displacement, cultural suppression, racism, and institutional misrecognition (Auger, 2016; Juutilainen et al., 2014; Nutton and Fast, 2015). This is why the model avoids treating psychological well-being as a purely internal condition. The risks under consideration emerge from damaged or pressured relations among self, community, land, language, and memory.

The fourth component is Indigenous oral tradition. The model conceptualizes oral traditions as living psychosocial knowledge embedded in community-held epistemic and relational infrastructures. Their epistemic role lies in organizing and transmitting knowledge concerning memory, ethics, identity, spirituality, land, and community life. Their relational role lies in sustaining connections among persons, generations, ancestors, language, place, and collective memory. Within these infrastructures, oral traditions also function as cultural resources whose relevance to well-being may be expressed through the four proposed pathways. Storytelling, creation accounts and sacred histories, songs, chants, ritual narratives, place-based narratives, oral histories, and ancestral accounts can transmit memory, identity, ethics, ecological knowledge, and belonging across generations (Mahuika, 2021; Mukuni, 2025; Struthers and Peden-McAlpine, 2005). They help communities interpret experience, sustain relations, and keep cultural worlds intelligible in changing conditions. The oral forms included in the model are illustrative categories rather than equivalent units of analysis. The model does not assume that storytelling, song, ritual narrative, oral history, or place-based accounts carry the same authority, access conditions, cultural functions, or psychosocial relevance across communities.

The fifth component consists of four proposed psychosocial pathways: meaning-making, cultural continuity, relational selfhood, and social cohesion. Through meaning-making, oral traditions may help individuals and communities interpret suffering, grief, displacement, or trauma within wider histories of survival and collective memory. Through cultural continuity, they connect past, present, and future, making identity more than a response to present-day external recognition. Through relational selfhood, they reground self-worth in relations with land, ancestors, language, spirituality, and community rather than in productivity, market value, or dominant institutional approval. Through social cohesion, they gather people into shared narration, listening, ritual, and memory, thereby strengthening belonging and support. These pathways are analytically distinct but practically intertwined. A song, for example, may be relevant to cultural continuity and social cohesion, while a place-based story may participate in meaning-making and relational self-understanding. These examples illustrate conceptual overlap rather than empirically established effects. As detailed in Section 6.6, the pathways differ in evidentiary status. The link between oral traditions and relational selfhood has limited context-specific support, while much of the available evidence concerns broader relations with land, family, community, culture, and self-determination rather than oral traditions themselves.

The final component is culturally grounded psychological well-being. It refers to the experienced capacity to sustain identity security, belonging, self-continuity, cultural connectedness, and a meaningful location within relations, histories, and obligations. These dimensions connect personal experience with relational, cultural, and community life. In the model, they serve as experiential expressions of well-being informed by Indigenous well-being research (Rountree and Smith, 2016; Sangha et al., 2024; Snowshoe et al., 2015).

The indicators are analytically distinguished from the four pathways. Within the model, pathway is the common organizing label for four concepts with different analytical functions. Meaning-making and social cohesion concern processes, cultural continuity concerns a temporal and cultural condition, and relational selfhood concerns a mode of personhood and self-understanding. Identity security, belonging, self-continuity, and cultural connectedness describe experiential dimensions of culturally grounded psychological well-being. Cultural continuity operates as a broader cultural condition and proposed pathway, whereas self-continuity refers to an experienced sense of connection across time. Collective resilience and community well-being are situated at the community level. The model therefore links experiential and collective dimensions within a relational field, consistent with Indigenous frameworks that understand individual, relational, cultural, and collective well-being as interdependent (Auger, 2016; Klassen et al., 2024; Martínez-Martínez et al., 2026; Ullrich, 2019).

**Figure 1:**
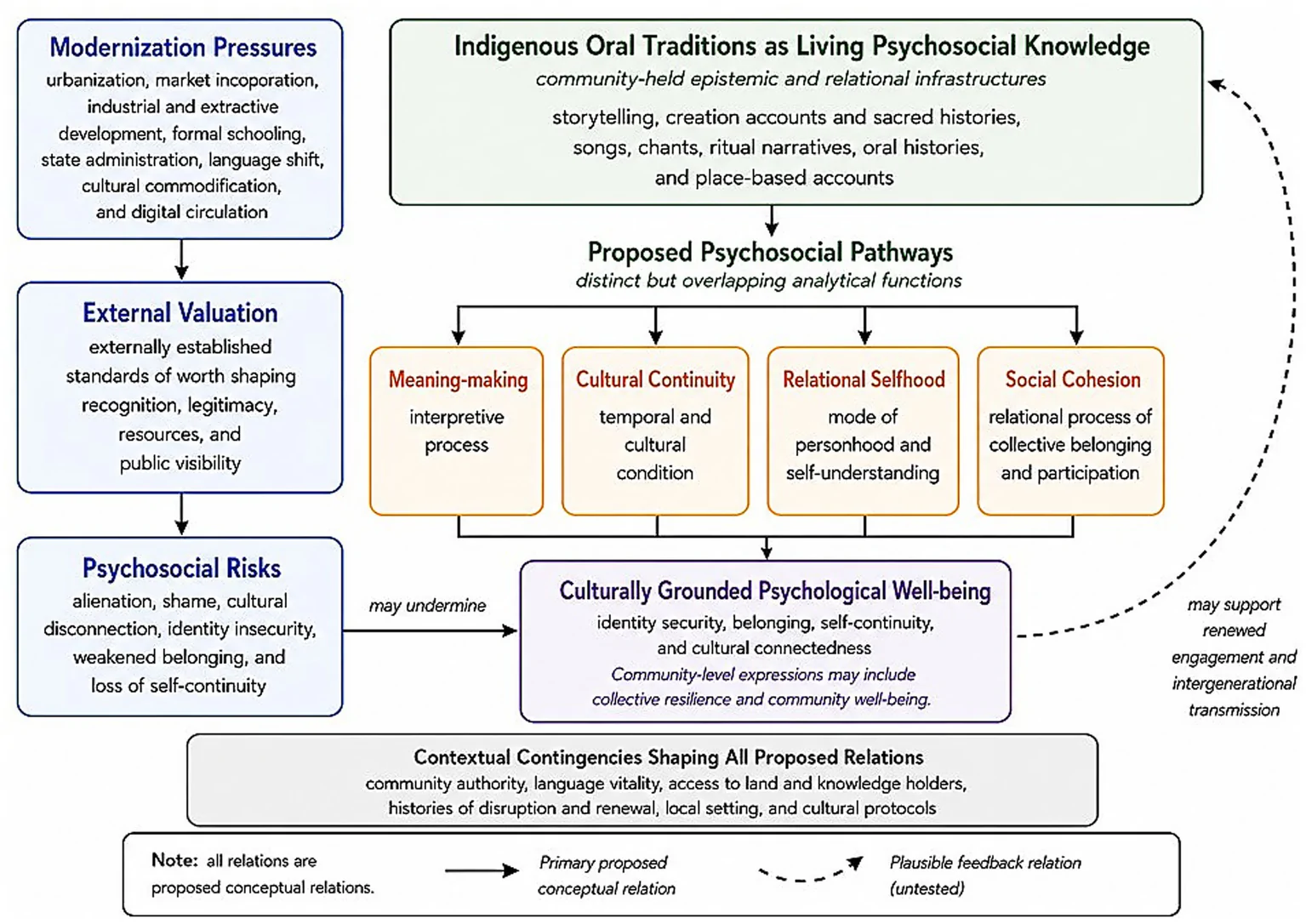
A conceptual model of Indigenous oral traditions and well-being. The model distinguishes modernization pressures, external valuation, psychosocial risks, Indigenous oral traditions, four proposed psychosocial pathways, and culturally grounded psychological well-being. Indigenous oral traditions are conceptualized as living psychosocial knowledge embedded in community-held epistemic and relational infrastructures. Meaning-making, cultural continuity, relational selfhood, and social cohesion are presented as four proposed pathways with distinct but overlapping analytical functions. The figure does not assign them equivalent causal roles. Solid arrows indicate primary proposed conceptual relations and do not represent a fixed, universal, or temporal causal sequence. The dashed arrow represents a plausible but untested feedback relation through which psychological well-being may support renewed engagement with oral traditions and intergenerational transmission. All relations are shaped by community authority, language vitality, access to land and knowledge holders, histories of disruption and renewal, local setting, and cultural protocols. The oral forms shown are illustrative and are not assumed to be interchangeable across communities or settings.

The model should not be read as a fixed sequence. It juxtaposes a risk relation and a culturally grounded support relation; it does not present oral traditions as a response caused by psychosocial risk or as a remedy that necessarily follows it. Modernization pressures and external valuation are positioned as contextual and evaluative conditions, while psychosocial risks describe possible consequences rather than inevitable outcomes. Indigenous oral traditions are treated as living psychosocial knowledge, and the four pathways identify distinct analytical functions through which such knowledge may become relevant to culturally grounded well-being. These relations are context-dependent and shaped by community authority, language vitality, access to land and knowledge holders, urban or rural location, histories of disruption and renewal, and local rules governing transmission. The arrows in Figure 1 therefore represent conceptual relations for context-specific empirical examination rather than established causal effects. Figure 1 also includes a plausible feedback relation from psychological well-being to renewed engagement with oral traditions. The proposed relation reflects the possibility that greater identity security, belonging, self-continuity, and cultural connectedness may support continued participation in oral practices and strengthen intergenerational transmission. This feedback relation is theoretically plausible but remains empirically untested. It is therefore included to represent the potentially recursive character of the model rather than as an established causal effect.

The value of the model lies in making visible a conceptual relationship that is often acknowledged but not fully specified. Indigenous oral traditions are frequently described as culturally important, yet their relevance to psychological well-being is sometimes left at the level of general affirmation. By identifying specific psychosocial pathways, the model offers a more precise way to understand that relevance. It shows how oral traditions may matter not only because they preserve heritage, but because they sustain the meanings, relations, and continuities through which well-being becomes possible. The discussion that follows considers the theoretical, practical, and ethical implications of this model.

## 8 Discussion

The conceptual model developed in this article clarifies the psychological significance of Indigenous oral traditions as living, community-held knowledge embedded in cultural and relational life. Through stories, songs, ritual narratives, ancestral accounts, and place-based memory, Indigenous communities continue to organize meaning, belonging, continuity, and self-understanding. Their significance lies not only in preserving memory, identity, and intergenerational knowledge, but in sustaining the relational conditions through which well-being becomes intelligible.

This reframing is important because Indigenous psychological well-being is often shaped by the tension between external valuation and relational selfhood. Modernization introduces new institutions, economic demands, educational systems, and forms of public recognition. It also reshapes the criteria through which people, languages, places, and cultural practices are valued. When Indigenous knowledge is recognized mainly for its marketability, policy relevance, educational measurability, or public visibility, other forms of value may become less visible. Oral traditions offer another ground of meaning. They place the self within relations of land, ancestry, language, spirituality, memory, and community, and in doing so they expand the bases through which self-worth and collective worth can be understood. External valuation is used here in this limited analytical sense. It does not subsume coloniality, epistemic injustice, structural racism, assimilation pressure, misrecognition, symbolic violence, or cultural commodification. Instead, it isolates the evaluative moment through which broader relations of power may be translated into institutional judgments about legitimacy and worth (Damoah et al., 2024; Kearney, 2021; Lewis et al., 2025).

What the model adds is a more explicit account of this relationship. Indigenous oral traditions are often acknowledged as culturally significant, yet the connection between oral tradition and psychological well-being can remain broadly stated. The four pathways proposed in this article—meaning-making, cultural continuity, relational selfhood, and social cohesion—offer a more precise vocabulary for describing this connection. They distinguish several ways in which oral traditions may be conceptually related to narratable suffering, continuity across generations, relational self-understanding, shared memory, and belonging. The evidentiary basis for these relations is uneven. Cultural continuity is supported by a comparatively developed body of Indigenous health and well-being research, although much of this evidence concerns culture and connectedness broadly rather than oral traditions alone. Meaning-making and social cohesion are supported mainly by qualitative and context-specific work. Relational selfhood has limited direct support from particular oral or story-based practices and broader indirect support from research on land, reconnection, family, community, intergenerational continuity, and self-determination, but the specific contribution of oral traditions remains insufficiently tested. These pathways are analytically distinguishable, but in lived contexts they are often intertwined. A song, story, ritual narrative, or place-based account may carry several of these functions at once.

The framework also clarifies the scope of psychological well-being. Psychological well-being is not defined here solely through emotional stability, individual adjustment, or symptom reduction. It is understood as an experienced outcome indicated by identity security, belonging, self-continuity, cultural connectedness, and the capacity to locate one’s life within meaningful relations, histories, and obligations. Indigenous well-being scholarship supports the need for relational, cultural, spiritual, and strengths-based dimensions that are not adequately represented by narrowly individualized measures (Le Grande et al., 2017; Rountree and Smith, 2016; Sangha et al., 2024; Snowshoe et al., 2015). At the same time, the model does not collapse determinants, pathways, indicators, and collective outcomes into a single category. Meaning-making, cultural continuity, relational selfhood, and social cohesion are retained as proposed pathways with different analytical functions: two concern processes, one concerns a temporal and cultural condition, and one concerns a mode of relational personhood and self-understanding. Identity security, belonging, self-continuity, and cultural connectedness indicate how well-being may be experienced. Collective resilience and community well-being refer to a broader level of analysis. These distinctions are analytical rather than ontological: they clarify the structure of the model without denying the interdependence of individual, relational, cultural, and community life.

The model has practical implications for culturally inclusive mental health work. If oral traditions are living psychosocial knowledge, then they should be engaged as community-held knowledge rather than treated as material detached from community contexts. Adding stories, rituals, songs, or symbols to an existing intervention may increase cultural visibility, but cultural visibility alone is not the same as cultural safety. Cultural safety depends on who defines the problem, who authorizes the use of knowledge, who controls its circulation and reuse, who determines acceptable forms of benefit, and whether the meanings of oral traditions remain accountable to the communities that hold them.

For this reason, any research or mental health initiative engaging Indigenous oral traditions should begin with community authority and locally defined cultural protocols. Decisions about whether particular knowledge may be recorded, adapted, interpreted, circulated, or reused should remain with the relevant community and its recognized knowledge holders. Community-led governance, Indigenous data sovereignty, and community-defined benefit are therefore conditions of legitimate engagement rather than supplementary ethical considerations (Carroll et al., 2025; Chigwada et al., 2026; Chung-Do et al., 2022; Kurtz et al., 2024; Prehn et al., 2025).

Where knowledge is collectively held, individual consent alone may be insufficient, and the appropriate form of collective authorization should be determined within the community concerned. Elders, language holders, ceremonial authorities, and other knowledge custodians should be involved where local protocols assign them responsibility. The same principle applies to decisions about access, storage, attribution, future use, and public dissemination. These requirements should not be treated as a universal protocol, because authority and conditions of sharing differ across Indigenous communities and forms of knowledge (Datta et al., 2026; Kurtz et al., 2024). Any empirical development or practical application of the framework should therefore be undertaken with the relevant community and under locally recognized authorities and cultural protocols.

This perspective also places psychological well-being within a broader relational field. Although collective resilience is not treated as an individual indicator in the model, Indigenous resilience may involve relations with land, ancestors, language, community, spirituality, and collective memory. Oral traditions may be relevant to the continuation and renewal of these relations through remembering, gathering, teaching, and transmission. The framework therefore situates individual experience within wider relational and community processes without treating collective resilience as equivalent to psychological well-being.

### 8.1 Limitations

This conceptual analysis identifies cross-contextual relations in published scholarship and provides a framework for community-specific inquiry. Nation-specific histories, languages, territories, laws, authorities, ceremonial relations, and protocols remain central to interpretation. The evidence base includes broader research on Indigenous well-being, community-specific studies of oral practices, and adjacent conceptual scholarship, with each category assigned a distinct role in the synthesis. Sacred, restricted, and orally governed knowledge remains subject to community authority and conditions of access. The framework serves as a guide for examining how particular oral practices are understood, governed, renewed, and related to well-being in particular settings. Future research can develop community-defined indicators and evaluate the relations proposed in the model through locally authorized methods.

### 8.2 Directions for future research

The scope of this article points to several directions for future research. The next step is to examine how the proposed pathways operate in specific Indigenous communities and cultural settings. Community-based participatory research could explore how community members define the relationship between oral traditions and well-being. Ethnographic and narrative methods could examine how stories, songs, rituals, and place-based accounts relate to self-understanding, belonging, and continuity in everyday life. Longitudinal research could investigate whether cultural continuity, self-continuity, or cultural connectedness help explain associations between engagement with oral traditions and well-being. Comparative research may also examine how these relations vary across urban, rural, and land-based settings without treating communities as interchangeable.

Such future work would benefit from close attention to local authority, language, protocol, and historical context. Indigenous communities differ in their forms of oral tradition, their histories of cultural disruption and renewal, and their rules about what may be shared, who may speak, and how knowledge should be transmitted. These differences are not obstacles to theory-building. They are part of the living character of oral traditions. A framework attentive to this diversity can avoid treating oral traditions as uniform cultural objects while still recognizing their broader psychosocial significance. In this sense, the value of oral traditions for psychological well-being lies not only in what they preserve, but in the relational worlds they continue to sustain.
